# Evaluation of Insulin Medium or Chondrogenic Medium on Proliferation and Chondrogenesis of ATDC5 Cells

**DOI:** 10.1155/2014/569241

**Published:** 2014-04-10

**Authors:** Yongchang Yao, Zhichen Zhai, Yingjun Wang

**Affiliations:** ^1^School of Materials Science and Engineering, South China University of Technology, Guangzhou 510641, China; ^2^National Engineering Research Center for Tissue Restoration and Reconstruction, Guangzhou 510006, China

## Abstract

*Background*. The ATDC5 cell line is regarded as an excellent cell model for chondrogenesis. In most studies with ATDC5 cells, insulin medium (IM) was used to induce chondrogenesis while chondrogenic medium (CM), which was usually applied in chondrogenesis of mesenchymal stem cells (MSCs), was rarely used for ATDC5 cells. This study was mainly designed to investigate the effect of IM, CM, and growth medium (GM) on chondrogenesis of ATDC5 cells. *Methods*. ATDC5 cells were, respectively, cultured in IM, CM, and GM for a certain time. Then the proliferation and the chondrogenesis progress of cells in these groups were analyzed. *Results*. Compared with CM and GM, IM promoted the proliferation of cells significantly. CM was effective for enhancement of cartilage specific markers, while IM induced the cells to express endochondral ossification related genes. Although GAG deposition per cell in CM group was significantly higher than that in IM and GM groups, the total GAG contents in IM group were the most. *Conclusion*. This study demonstrated that CM focused on induction of chondrogenic differentiation while IM was in favor of promoting proliferation and expression of endochondral ossification related genes. Combinational use of these two media would be more beneficial to bone/cartilage repair.

## 1. Introduction

Cartilage is a very complex and avascular tissue, which would lead to the limited capacity for self-repair once cartilage is damaged. Tissue engineering and regenerative medicine provide an excellent way for cartilage repair. Although, in principle, autologous chondrocytes are the best cells for cartilage tissue engineering applications, it is difficult to acquire sufficient chondrocytes for tissue repair because of the damage to the donor, poor proliferation* in vitro*, and so on [1, 2]. In this context, stem cells including embryonic and mesenchymal cells appear as a promising alternative and are widely studied for cartilage regeneration [3].

The issue of how to induce the differentiation of stem cells efficiently and keep the long-lasting function is still not addressed. Differentiation capability of stem cells will be varied with different conditions, such as cell source and passage [4], causing different even conflicting results. For instance, TGF-*β*3, which is referred to as a chondrogenesis-inducing factor in most circumstances, may not promote chondrogenesis or even inhibit it in some cases [5, 6]. Therefore, researchers endeavored to look for another stable model cell line with such properties as indefinite and rapid proliferation as well as homogenous stability. Recently, lots of cell lines, such as C3H10T1/2 [7], ATDC5 [8], RCJ3.1C5 [9], CFK2 [10], C2C12 [11], MG63 [12], and MC3T3-E1 [13], have been widely used for the study of chondrogenesis and osteogenesis. ATDC5 was derived from AT805 teratocarcinoma cell line in 1990 by Atsumi et al. [8]. Since then, more and more studies have demonstrated that ATDC5 cell line had nearly the same characteristic of chondrogenesis as mesenchymal stem cell. As the ATDC5 cell line was superior in chondrocytic differentiation to C3H10T1/2 and RJC3.1 chondrogenic cell lines [8], it was well acknowledged as an* in vitro* chondrogenic model.

Insulin medium (IM) was used in most studies with ATDC5 cells for chondrocyte differentiation [7, 8, 14]. Tare et al. [15] reported that chondrogenic medium (CM), commonly used for chondrogenesis of MSCs and other stem cells, was effective for chondrocyte differentiation of ATDC5. In this study, ATDC5 cells were cultured in IM, CM, and growth medium (GM) to evaluate the effect of IM and CM on chondrogenesis of ATDC5 cells. Quantitative RT-PCR (qRT-PCR) and histological staining were performed to confirm chondrogenic differentiation of ATDC5.

## 2. Materials and Methods

### 2.1. Cell Culture

ATDC5 cells were cultured in GM containing 1 : 1 mixture of Dulbecco's modified Eagle's medium and Ham's F-12 medium supplemented with 5% fetal bovine serum (Invitrogen) in culture flasks at 37°C under 5% CO_2_. The culture medium was changed every 3 days.

### 2.2. Differentiation of ATDC5 Cells Induced by Different Medium

ATDC5 cells were cultured in 24-well plates with GM, IM, and CM for 21 days. The ingredients of the above-mentioned medium were shown as follows: CM consisted of high-glucose Dulbecco's modified Eagle medium (H-DMEM) (Gibco) supplemented with 10 ng/mL recombinant human transforming growth factor-*β*3 (TGF-*β*3) (Peprotech), 100 nM dexamethasone (Sigma), 50 *μ*g/mL ascorbic acid 2-phosphate (Sigma), 1 mM sodium pyruvate (Amersco), 40 *μ*g/mL proline (Biosharp), and ITS+ premix (BD; final concentrations: 6.25 *μ*g/mL bovine insulin, 6.25 *μ*g/mL transferrin, 6.25 *μ*g/mL selenous acid, 5.33 *μ*g/mL linoleic acid, and 1.25 mg/mL bovine serum albumin); IM was GM supplementing with 10 *μ*g/mL bovine insulin (Sigma). The density of cells in each well was 5 × 10^4^ cells.

For the experiment to select optimal TGF-*β*3 concentration for chondrogenesis of ATDC5 cells, the following concentrations of TGF-*β*3, 0, 2, 5, 10, 20, and 50 ng/mL, were used in CM, respectively. Cells with the same original density were cultured in 24-well plates for 14 days.

In all groups, medium was changed every 3 days.

### 2.3. Cell Proliferation Analysis

Cells in three groups were analyzed at various time points as indicated in the text and figure captions by CCK-8 and double-strand DNA (dsDNA) for cell quantification.

CCK-8 detection was preformed according to the manufacturer's instruction. Briefly, at each time point, cells were seeded at 96-well plate with 1000 cells per well followed by aspiration of the old medium and replacement with 110 *μ*L of fresh medium containing CCK-8 regent (Dojindo, premix 10 *μ*L of CCK-8 every 100 *μ*L of medium). After 2 hours of incubation at 37°C under 5% CO_2_, the absorbance for each sample was determined using a microplate reader set to 450 nm.

PicoGreen (Invitrogen) was used for dsDNA quantification. Cells at each time point were reacted with lysing liquid, which contained 50 mM Na_3_PO_4_, 20 mM N-acetyl cysteine, and 28 *μ*g/mL papain, for 16 h at 60°C. The lysate was centrifuged at 10000 g for 10 min at 4°C and the dsDNA concentration of the supernatant was measured by PicoGreen according to the manufacturer's protocol.

### 2.4. Quantitative Analysis of Glycosaminoglycan (GAG) Synthesis

The GAG content was measured using the 1,9-dimethylmethylene blue method [16]. After being cultured under different medium for 21 days, cells were lysed by lysing liquid as described in dsDNA quantification. The 1,9-dimethylmethylene blue (Sigma) colorimetric assay was performed with chondroitin-6-sulfate (Sigma) as a standard. The results of GAGs were normalized to dsDNA content.

### 2.5. Real-Time PCR (qRT-PCR) Analysis

Total RNA was isolated at various time points as indicated in the text and figure captions using TRIzol Reagent (Invitrogen) according to the manufacturer's protocol. The RNA concentration was determined using a NanoDrop2000 spectrophotometer (Thermo Scientific) and reverse transcription reactions were performed from 500 ng of total RNA using a first cDNA synthesis kit (Fermentas). Real-time PCR reactions were conducted using SYBR green reagent (Invitrogen).

Primer sequences were listed in Table 1. Real-time PCR reactions were performed using the Chromo4 real-time PCR system (Bio-Rad). Samples were held at 95°C for 10 min, followed by 40 amplification cycles consisting of a denaturation step at 95°C for 15 s and an annealing and extension step at 60°C for 1 min. The threshold cycle values of the gene were normalized against Hprt and Ppia. Then the relative fold change was obtained by normalizing the data of each group against control group.

### 2.6. Alcian Blue Staining

After 21 days of culture, cells were fixed in 10% neutral buffered formalin and washed twice with PBS. Alcian Blue (Biosharp) staining was performed to detect proteoglycan. Samples were stained with 1% Alcian Blue 8GS (Fluka) in 0.1 MHCl for 5 min at room temperature.

### 2.7. Statistical Analysis

Repetitive ANOVA and Tukey's multiple comparison tests were used to determine statistical significance (*P* < 0.05) between groups. Experiments were repeated with *n* = 3 biological replicates and the results were represented as the mean ± standard deviation.

## 3. Results

### 3.1. Cell Proliferation in GM, IM, and CM

**Figure 1:**
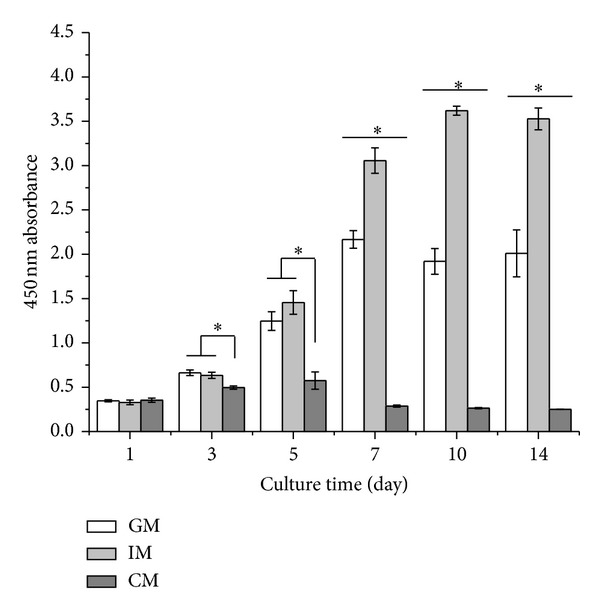
Proliferation of cells cultured in different media. Cells were cultured in GM, IM, and CM for 14 days. At each time point, cell number was determined using CCK-8 cell proliferation/cell viability kit. The amount of cells would be proportional to the absorbance at 450 nm. The results were presented as the means ± standard deviation (*n* = 6). *Significant difference (*P* < 0.05) between groups at same time point.

**Figure 2:**
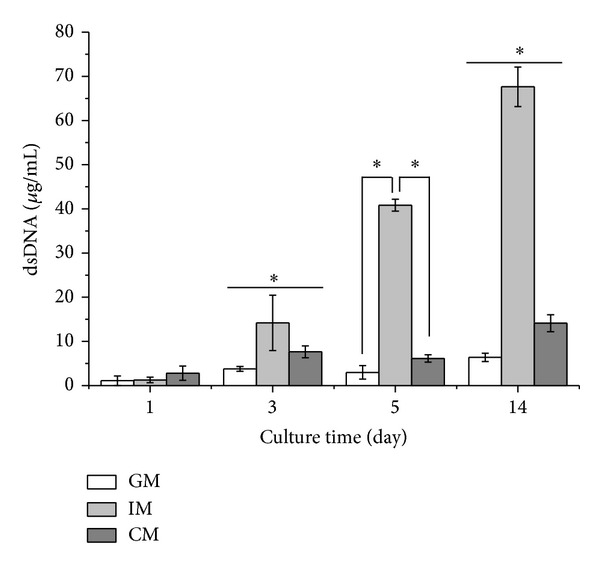
dsDNA quantification of cells cultured in different media. Cells were cultured in GM, IM, and CM for 14 days. At each time point, dsDNA content was detected by PicoGreen dsDNA kit. The amount of dsDNA content was directly in proportion to the cell number. The data were presented as the means ± standard deviation (*n* = 4). *Significant difference (*P* < 0.05) between groups at same time point.

**Figure 3:**
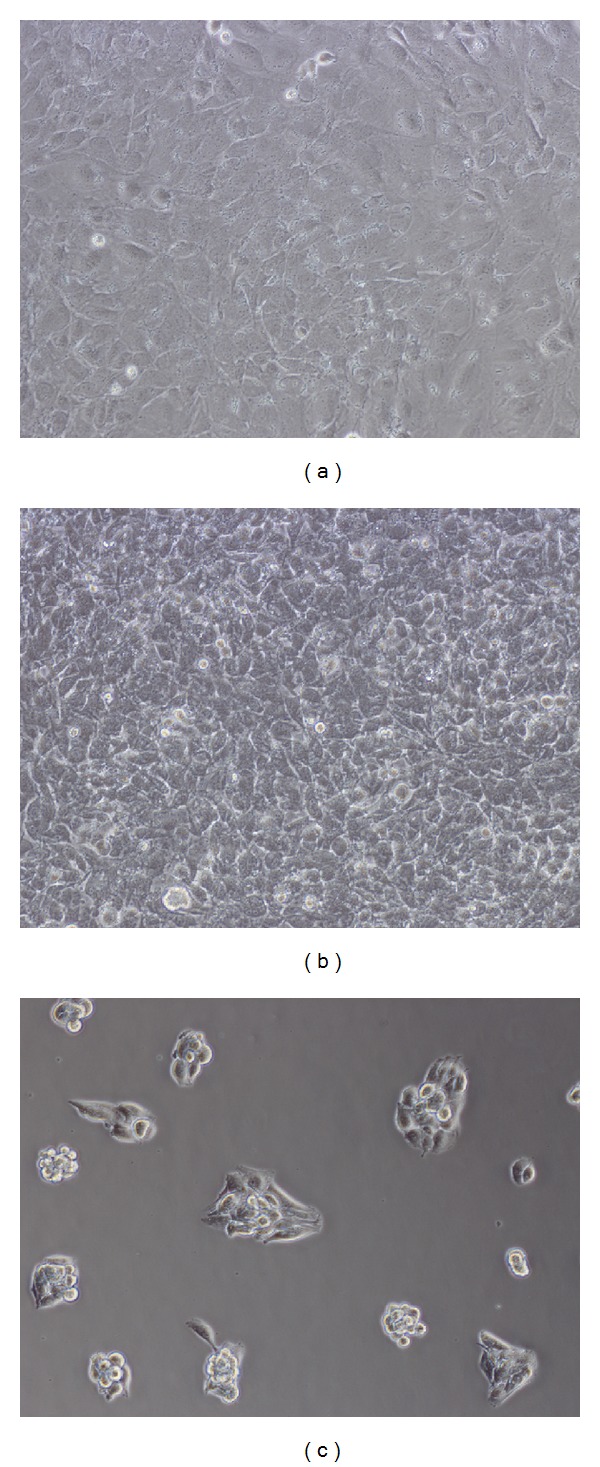
Morphology of ATDC5 cells cultured in (a) GM, (b) IM, and (c) CM at day 5. The pictures were taken under light microscope with 10x magnification.

ATDC5 cells were cultured in GM, IM, and CM for 14 days. At various time points, the amount of cells in different groups was determined using CCK-8 kit and PicoGreen dsDNA kit. As shown in Figure 1, at the beginning of cell culture, cells in all the three groups showed almost the same proliferation rate. With time, cells in IM group grew rapidly and exhibited the highest proliferation rate among the three groups. The amount of cells in IM group was maintained after day 10. Cells in GM groups also kept proliferating at a moderate speed until day 7 while the cells in CM group grew at the lowest rate and ceased proliferation after day 5. The data of dsDNA quantification shown in Figure 2 indicated a similar result. The quantity of dsDNA content in IM group was the highest among all the groups at day 3, day 5, and day 14, suggesting that IM promoted the proliferation of cells significantly. These data were consistent with the morphology of cells observed under the light microscope (Figure 3). At day 5, cells in IM and GM appeared about 90% confluence while those in CM gathered in to several rounded clumps.

### 3.2. Gene Expression Analysis in GM, IM, and CM

**Figure 4:**
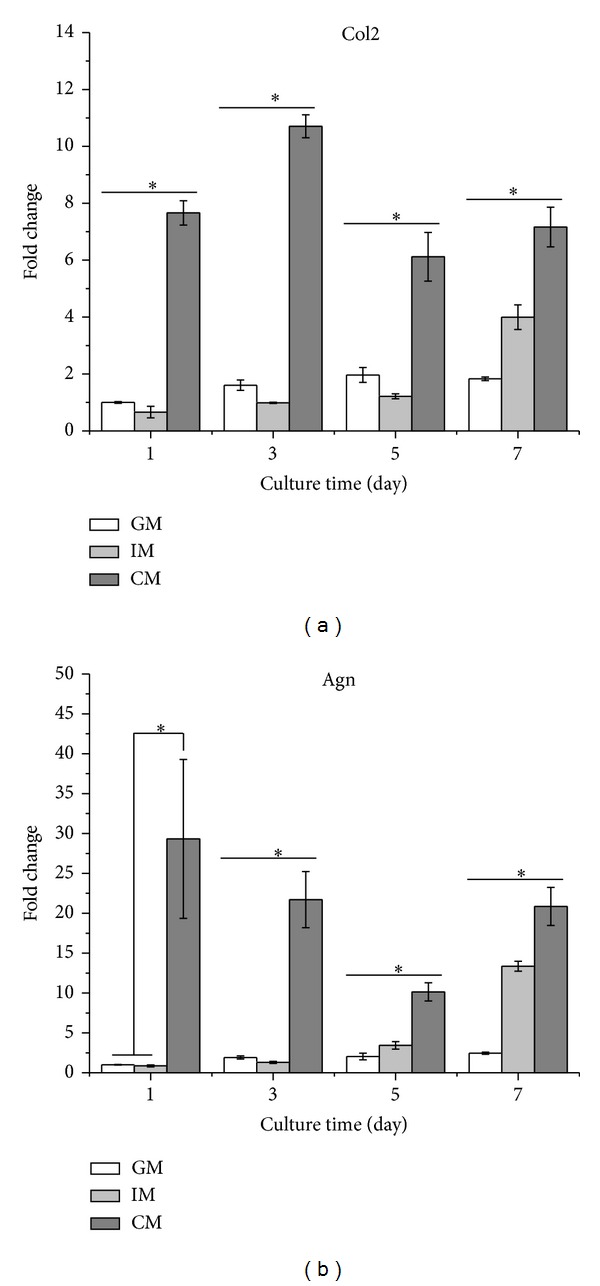
Expression level of chondrocyte specific genes of ATDC5 cells. Cells were cultured in CM and IM for 7 days. At the indicated time point, total RNA in all the three groups was isolated using TRIzol followed by PCR assay to evaluate the expression level of (a) Col2 and (b) Agn. The results were presented as the means ± standard deviation (*n* = 3).*Significant difference (*P* < 0.05) versus GM group.

Collagen type II (Col2) and aggrecan (Agn), cartilage specific markers, were putatively used to evaluate the degree of chondrocyte differentiation. As shown in Figure 4, ATDC5 cells cultured in CM exhibited much higher expression level of Col2 and Agn from day 1 to day 7 compared with those cultured in IM and GM. Cells in IM group expressed Col2 and Agn in a similar level to those in GM from day 1 to day 5. At day 7, there appeared more Col2 and Agn expression in IM group than that in GM group.

**Figure 5:**
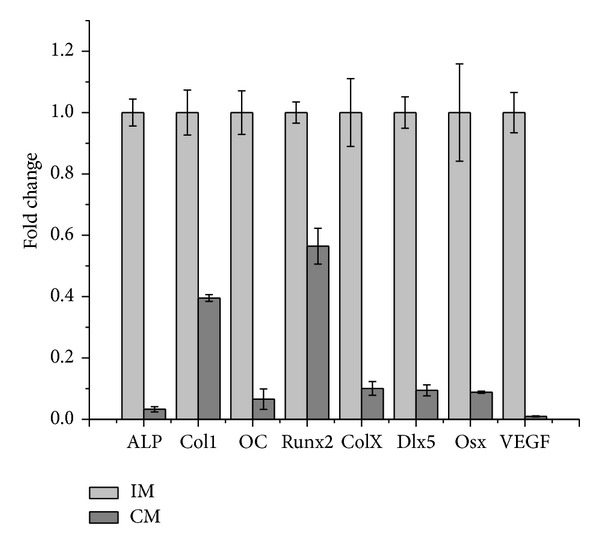
Expression level of endochondral ossification related genes of ATDC5 cells induced in IM and CM at day 14. The results were presented as the means ± standard deviation (*n* = 3). Expression fold change of all genes had significant difference (*P* < 0.05) between IM and CM.

Additionally, the expression level of hypertrophic marker genes including collagen type X (Col10), alkaline phosphatase (ALP), collagen type I (Col1), vascular endothelial growth factor (VEGF), and osteogenic target transcription factors Dlx5, Runx2, osterix (Osx), and osteocalcin (OC) were determined using qRT-PCR and compared between IM and CM groups (Figure 5). The data demonstrated that IM promoted the expression of all the above-mentioned genes of ATDC5 cells in comparison with CM.

### 3.3. Analysis of GAG Deposition

**Figure 6:**
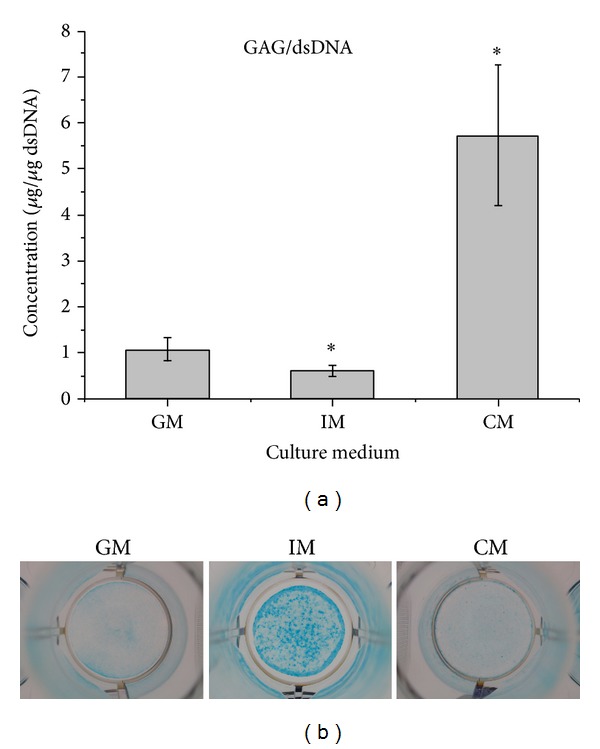
Analysis of GAG deposition of ATDC5 cells cultured in GM, IM, and CM for 21 d. (a) Quantitative analysis of GAGs synthesis using the 1,9-dimethylmethylene blue method. (b) Alcian Blue staining. The results were presented as the means ± standard deviation (*n* = 6). There was significant difference (*P* < 0.05) versus GM group.

GAG is a typical component of cartilaginous ECM. Our results showed that the level of GAG deposition per cell in CM group was significantly higher than that in GM and IM (Figure 6(a)). Interestingly, the total GAG contents of cells cultured in IM were the most among all the three groups, as evidenced by the most intensive staining shown in Figure 6(b).

### 3.4. Selection for TGF-*β*3 Concentration

**Figure 7:**
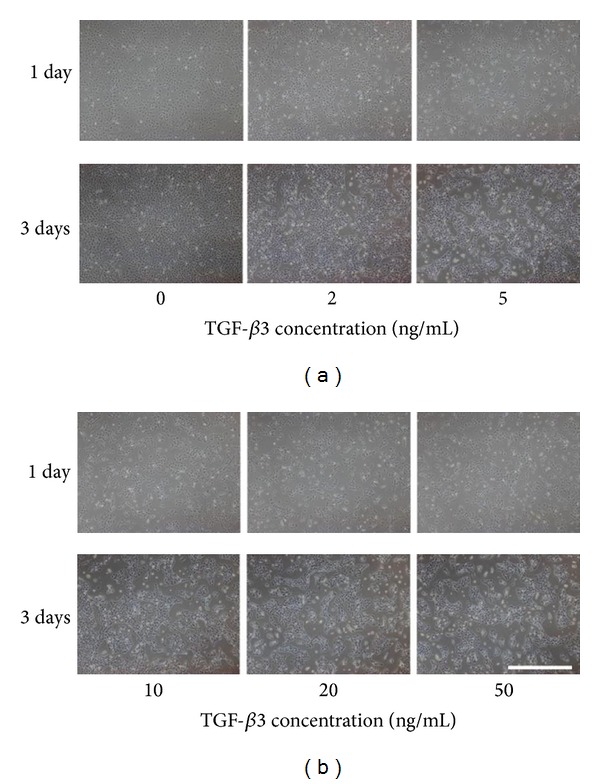
Cell morphology of ATDC5 cells cultured in CM with different TGF-*β*3 concentration at day 3. Bar = 500 *μ*m.

**Figure 8:**
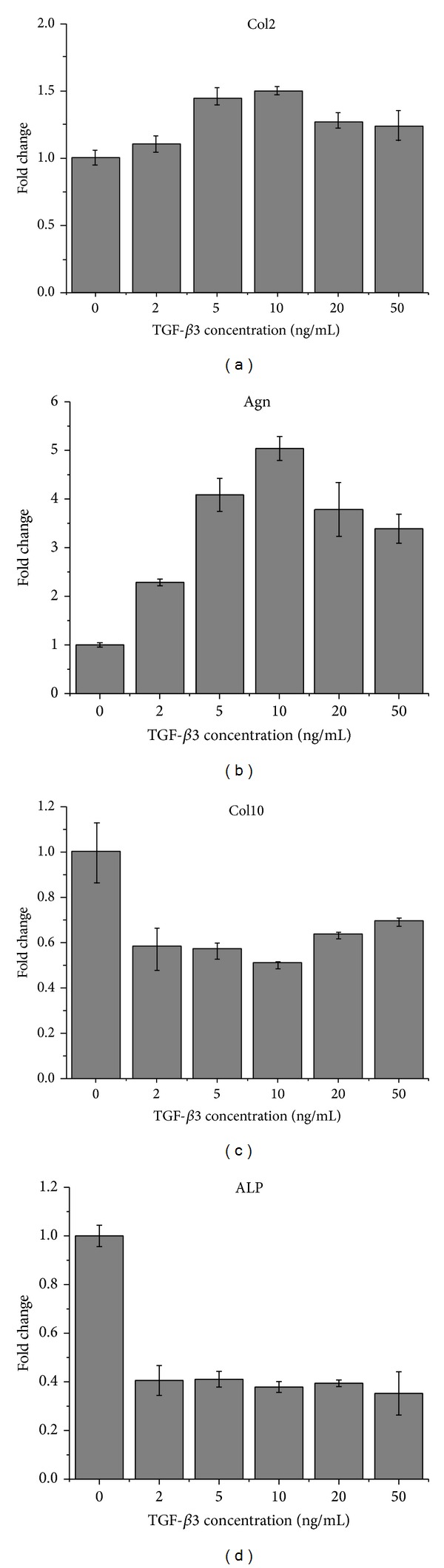
Gene expression level of ATDC5 cells cultured in CM with different TGF-*β*3 concentration at day 14. The results were presented as the means ± standard deviation (*n* = 3).

It was shown in Figure 7 that cells appeared agglomerate with escalating concentration of TGF-*β*3 at day 3. The data of qPCR (Figure 8) indicated that the expression level of Col2 and Agn in 10 ng/mL of TGF-*β*3 was superior to that in other concentrations of TGF-*β*3. On the other hand, 2 ng/mL TGF-*β*3 was sufficient to inhibit the expression of Col10 and ALP, while the inhibition effects showed no significant difference with the increasing TGF-*β*3 concentration. Taken together with all these data, 10 ng/mL was regarded as an optimal concentration to induce the chondrogenesis and prohibit the formation of hypertrophic chondrocyte.

## 4. Discussion

At present, people pin their hopes on stem cells with tissue engineering technology to cure osteochondral defect. However, numerous issues are still interfering with researchers. A lot of factors such as cell source [17–19], culture conditions [20–22], and stress [23–25], have various effects on osteochondral differentiation. Therefore, it is necessary to set up a model system to find out the optimal parameters so that suitable microenvironment would be established for chondrogenesis of stem cells. Among various kinds of cell lines, the ATDC5 cell line has attracted much more attention as a model cell line for chondrocyte differentiation studies because of its excellent characteristics including easy culture, rapid proliferation, and maintenance of undiferentiation and homogeneity under normal culture conditions [26].

In this study, to investigate the effect of different medium on osteochondral differentiation, ATDC5 cells were cultured in monolayer which would remove the influences of the morphological and structural differences of scaffolds. The data of CCK-8 assay and dsDNA content test (shown in Figures 1 and 2) demonstrated that IM significantly accelerated the proliferation rate of cells. Cells cultured in CM were inclined to agglomerate at a slow growth rate (Figure 3). It was shown in Figure 4 that the expression of cartilage markers (Col2 and Agn) in CM group was remarkably enhanced at day 1 compared with GM group, whereas there was no obvious difference for expression level of Col2 and Agn between GM and IM until day 7. This result suggested that CM induced the chondrogenesis of ATDC5 cells sooner than IM did. And owing to the decrease of cell proliferation by differentiation process, cells in CM group grew much slower than those in IM group. Moreover, CM was favorable for inhibition of hypertrophy while IM promoted the expression of endochondral ossification related genes (Figure 5). And it was interesting that the level of GAG deposition per cell in CM group was the highest among all the groups whereas the greatest quantity of total GAG belonged to IM group, which was attributed to the vast amount of cells in IM group.

In addition, the preferable concentration of TGF-*β*3 to obtain effective chondrocyte differentiation and avoid hypertrophy was selected as 10 ng/mL using ATDC 5 cells. TGF-*β*3 could promote chondrocytic differentiation when in the right concentration. There would be no significant effect on chondrogenesis when too low TGF-*β*3 concentration was applied. However, excessive supply of TGF-*β*3 would not be effective in inducing chondrogenesis either. TGF beta could promote BMP signaling pathways while BMP2 significantly decreased TGF signaling pathways [27]. Buxton et al. found that temporal exposure of chondrogenic factor would lead to more production of cartilaginous matrix than continuous exposure did [28]. Therefore, neither the higher nor the longer treatment of growth factor would be beneficial for chondrogenesis. But studies are needed to figure out the optimal way to deliver chondrogenic factors. In addition, TGF beta could inhibit hypertrophy of cells and osteochondral ossification [29]. Low concentration of TGF beta could restrain the expression of Col10 and ALP. But with the increase of concentration, no obvious change appeared. And the data was consistent with other studies for chondrogenesis of mesenchymal stem cells (MSCs), suggesting the result obtained from study of ATDC 5 cells could apply to that of MSCs too.

A suitable cell density is required for chondrocytic differentiation. To some extent, higher density of cells was more beneficial for chondrogenesis than lower density did. Based on the above results, we believed IM and CM could be combined to induce the chondrogenesis for better outcome. IM would be used first to accelerate the cell growth and then would be replaced by CM for chondrocyte differentiation and suppression of endochondral ossification. More work would be needed to find out the suitable parameters.

Lots of studies have used ATDC 5 cells to explore the influence of other factors on chondrogenesis, including laser irradiation [30], oxygen [31, 32], and mechanical interaction [33, 34]. Besides chondrogenesis, ATDC 5 cells would be also used as model cells for endochondral ossification [35]. With the use of stable model system, some parameters such as suitable seeding densities and selection of biological molecules would be optimized and the underlying mechanisms involved in the process of endochondral ossification would be elucidated. Thus, with gradual understanding of influence of various factors on endochondral ossification, favorable microenvironment could be established for bone/cartilage repair.

## Figures and Tables

**Table 1:** Primers used in this study.

Primer	Forward sequence	Reverse sequence	Amplicon	Access number
Hprt	CTGGTGAAAAGGACCTCTCGAA	CTGAAGTACTCATTATAGTCAAGGGCAT	110	NM_013556.2
Ppia	CGCGTCTCCTTCGAGCTGTTTG	TGTAAAGTCACCACCCTGGCACAT	150	NM_008907.1
Col2	AGGGCAACAGCAGGTTCACATAC	TGTCCACACCAAATTCCTGTTCA	171	NM_031163.3
Agn	AGTGGATCGGTCTGAATGACAGG	AGAAGTTGTCAGGCTGGTTTGGA	105	NM_007424.2
Col1	ATGCCGCGACCTCAAGATG	TGAGGCACAGACGGCTGAGTA	153	NM_007742.3
ColX	TTCTGCTGCTAATGTTCTTGACC	GGGATGAAGTATTGTGTCTTGGG	115	NM_009925.4
ALP	TGCCTACTTGTGTGGCGTGAA	TCACCCGAGTGGTAGTCACAATG	164	NM_007431.2
OC	AGCAGCTTGGCCCAGACCTA	TAGCGCCGGAGTCTGTTCACTAC	178	NM_007541.2
Runx2	CACTGGCGGTGCAACAAGA	TTTCATAACAGCGGAGGCATTTC	144	NM_009820.4
Dlx5	TACAACCGCGTCCCGAGT	AATAGTCCTGGGTTTACGAA	108	NM_010056.2
Osx	CGTCCTCTCTGCTTGAGGAA	CTTGAGAAGGGAGCTGGGTA	196	NM_130458.3
VEGF	ACGCATTCCCGGGCAGGTGAC	TCTTCCGGGCTTGGCGATTTAG	93	NM_009505.4
